# Evidence against roles for phorbol binding protein Munc13-1, ADAM adaptor Eve-1, or vesicle trafficking phosphoproteins Munc18 or NSF as phospho-state-sensitive modulators of phorbol/PKC-activated Alzheimer APP ectodomain shedding

**DOI:** 10.1186/1750-1326-2-23

**Published:** 2007-12-09

**Authors:** Annat F Ikin, Mirsada Causevic, Steve Pedrini, Lyndsey S Benson, Joseph D Buxbaum, Toshiharu Suzuki, Simon Lovestone, Shigeki Higashiyama, Tomas Mustelin, Robert D Burgoyne, Sam Gandy

**Affiliations:** 1 Farber Institute for Neurosciences of Thomas Jefferson University, 900 Walnut Street, Philadelphia, 19107, PA, USA; 2 Mount Sinai School of Medicine, One Gustave L. Levy Place, New York, 10029, NY, USA; 3 Hokkaido University, Sapporo, Japan; 4 Ehime University School of Medicine, Ehime, Japan; 5 The Burnham Institute, La Jolla CA, USA; 6 Physiological Laboratory, University of Liverpool, Crown St, Liverpool, L69 3BX, UK

## Abstract

**Background:**

Shedding of the Alzheimer amyloid precursor protein (APP) ectodomain can be accelerated by phorbol esters, compounds that act via protein kinase C (PKC) or through unconventional phorbol-binding proteins such as Munc13-1. We have previously demonstrated that application of phorbol esters or purified PKC potentiates budding of APP-bearing secretory vesicles at the *trans*-Golgi network (TGN) and toward the plasma membrane where APP becomes a substrate for enzymes responsible for shedding, known collectively as α-secretase(s). However, molecular identification of the presumptive "phospho-state-sensitive modulators of ectodomain shedding" (PMES) responsible for regulated shedding has been challenging. Here, we examined the effects on APP ectodomain shedding of four phorbol-sensitive proteins involved in regulation of vesicular membrane trafficking of APP: Munc13-1, Munc18, NSF, and Eve-1.

**Results:**

Overexpression of either phorbol-sensitive wildtype Munc13-1 or phorbol-insensitive Munc13-1 H567K resulted in increased basal APP ectodomain shedding. However, in contrast to the report of Roßner *et al *(2004), phorbol ester-dependent APP ectodomain shedding from cells overexpressing APP and Munc13-1 wildtype was indistinguishable from that observed following application of phorbol to cells overexpressing APP and Munc13-1 H567K mutant. This pattern of similar effects on basal and stimulated APP shedding was also observed for Munc18 and NSF. Eve-1, an ADAM adaptor protein reported to be essential for PKC-regulated shedding of pro-EGF, was found to play no obvious role in regulated shedding of sAPPα.

**Conclusion:**

Our results indicate that, in the HEK293 system, Munc13-1, Munc18, NSF, and EVE-1 fail to meet essential criteria for identity as PMES for APP.

## Introduction

The main constituent of cerebral and cerebrovascular amyloid found in the brains of Alzheimer's disease patients is the amyloid-β peptide (Aβ). Aβ is derived from a 695/751/770 amino acid precursor, termed the amyloid precursor protein (APP) via a potentially amyloidogenic pathway (for review, see [1]). In this pathway, APP is first cleaved by BACE (**b**eta-site **A**PP-**c**leaving **e**nzyme) or β-secretase, that releases a large, extracellular portion called soluble APP or sAPPβ, followed by cleavage by a second enzyme, γ-secretase, that releases Aβ peptide and the cytoplasmic APP remnant called "AICD" (APP intracellular domain). An alternative APP processing pathway – the non-amyloidogenic pathway of APP proteolysis – precludes the production of the neurotoxic Aβ peptide. In this pathway, the enzyme α-secretase cleaves APP between residues K^16 ^and L^17 ^within the Aβ domain. This event releases a large, soluble extracellular fragment or sAPPα leaving a short, carboxyl-terminal fragment consisting of 83 amino acids (C83) associated with the cell membrane. γ-secretase then cleaves C83 generating a non-amyloidogenic, 3-kDa fragment called p3.

Protein phosphorylation mediated by protein kinase C (PKC) activates the proteolysis of APP by α-secretase causing an increase in shedding of the soluble APP ectodomain or sAPPα [2,3]. A number of enzymes can act as α-secretases. All are members of the ADAM (**a d**isintegrin **a**nd **m**etalloprotease domain) family, which is comprised of transmembrane proteins responsible for extracellular proteolysis of target proteins located on the cell surface or within the extracellular matrix. ADAM activity results in the ectodomain shedding of a number of substrates, including APP. ADAM proteins such as ADAM9, ADAM10 and ADAM17/TACE have been demonstrated to constitute a set of α-secretase enzymes that carry out either the basal (constitutive) or the PKC/phorbol ester-regulated proteolysis of APP [4-6], both at the plasma membrane and within the *trans*-Golgi network (TGN) [7,8]. We have previously demonstrated that application of phorbol 12,13-dibutyrate (PDBu) to intact cells or application of purified PKC to TGN-rich fractions increases the biogenesis of APP-bearing, secretory vesicles from the TGN [9]. Therefore, we hypothesized that one or more phorbol ester receptors/PKC substrates that are components of the universal transport vesicle machinery of the central vacuolar pathway (responsible for vesicle budding, scission, transport, priming and/or fusion) might play important roles in trafficking of APP through the secretory pathway, which conveys APP to the plasma membrane where α-secretases/ADAM enzymes are concentrated.

Munc13-1 was the first candidate APP shedding regulator that we considered. Munc13 (Murine homologue of *uncoordinated-13*) is the mammalian homologue of *C. elegans unc-13*. Munc13 is a novel, non-PKC, diacylglycerol (DAG)/phorbol ester receptor that is essential for vesicle priming at the active zone [10,11]. Munc13-1 is one of three brain-specific Munc13 isoforms [12]. Munc13-1 contains: an N-terminal Ca^2+^-binding or C2 domain; a C1 domain consisting of a high-affinity DAG/phorbol ester-binding site tandem to and a second C2 domain; two Munc13 homology domains (MHD1 and MHD2); and a third, C-terminal C2 domain. Munc13-1-mediated priming is stimulated by the binding of DAG/phorbol esters to the Munc13-1 C1 domain, followed by the translocation of the cytoplasmic Munc13-1 protein to the plasma membrane. A point mutation in the first histidine residue in the C1 domain of Munc13-1, an H567K mutation, prevents phorbol binding and, consequently, prevents plasma membrane re-localization of Munc13-1 and abolishes Munc13-1 vesicle priming activity. Most notably with regard to the current study, Munc13-1 C1 domain function (i.e., phorbol sensing) has been reported to control APP shedding [13].

Whilst Munc13 proteins regulate the priming step in the transport of synaptic vesicles, another protein and PKC target, Munc18-1 (Mammalian homologue of *S. cerevisiae *Sec1p and of *C. elegans uncoordinated-18*) has been demonstrated to control multiple steps in trafficking of membrane proteins through the constitutive secretory pathway. These include 1) vesicle docking, (i.e., tethering of vesicles to the plasma membrane prior to their priming for fusion), 2) priming *per se *(i.e., "maturation" of docked vesicles ready for fusion), and 3) fusion of vesicles with the plasma membrane. Munc18 is a SNARE (**s**oluble ***N***-ethylmaleimide-sensitive fusion protein [NSF]-**a**ttachment protein [SNAP] **re**ceptors) complex accessory protein that participates in the regulation of neurosecretion by interacting with a SNARE protein syntaxin-1A, an event that is incompletely understood [14,15]. However, it has been demonstrated that Munc18 is phosphorylated by PKC in neuronal and neuroendocrine or chromaffin cells and that Munc18 phosphorylation state regulates the spontaneous release of vesicular content by altering Munc18-syntaxin-1 interaction [16-18]. This latter property raised the question of whether Munc18 might be a PMES for APP.

The third candidate regulator of APP shedding that we considered was NSF itself. The tyrosine phosphatase PTP-MEG2 is targeted by its amino-terminal Sec14p homology domain to the membrane of secretory vesicles. There, PTP-MEG2 regulates vesicle size by promoting homotypic vesicle fusion by a mechanism that requires its catalytic activity. Huynh *et al *[19] identified NSF as a substrate for PTP-MEG2. Phosphorylation of NSF at Tyr 83, or an acidic substitution at the same site, can prevent α-SNAP binding. Conversely, expression of a Y83F mutant of NSF causes excessive spontaneous fusion events. Since such a mechanism could conceivably underlie phorbol-enhanced intracellular cleavage of APP by α-secretase [8], NSF was identified as another potential PMES for APP.

Finally, the fourth molecule that we considered was Eve-1, a protein identified in a two-hybrid screen using the ADAM12 cytoplasmic tail [20]. Eve-1 was discovered to be essential for phorbol-activated shedding of proEGF [20]. The proposed underlying mechanism in this case is that phosphorylation of Eve-1 (or another protein of the Eve-1/ADAM12 complex) dissociates ADAM12 from Eve-1 so that ADAM12 relocalizes to the plasma membrane where ADAM12 can cleave APP and shed the APP ectodomain. The essential nature of the Eve-1 role in regulated shedding of pro-EGF raised the question of whether Eve-1 might be a phosphoprotein and, if so, whether its phosphorylation state might modulate APP shedding.

In summary, in this study, we evaluated the roles of wildtype and C1 mutant Munc13-1, as well as the roles of wild type and phospho-site mutants of Munc18 and NSF. We also evaluated ADAM adaptor Eve-1 for its potential identity as a phosphoprotein and for its potential role as a PMES for APP.

## Results and Discussion

### Munc13-1

Our investigations into the role of Munc13-1 in the processing of the Alzheimer amyloid precursor protein (APP) were carried out in human embryonic kidney 293 cells. This non-neuronal cell line has been extensively used as a model system for analyses of different pathways and molecules that regulate APP metabolism [21-28]. We co-transfected HEK293 cells with cDNAs for human APP and either Munc13-1 wild type or Munc13-1 H567K mutant, and then studied the metabolism and sub-cellular distribution of APP in the absence or presence of phorbol ester 12-myristate 13-acetate (PMA).

**Figure 1:**
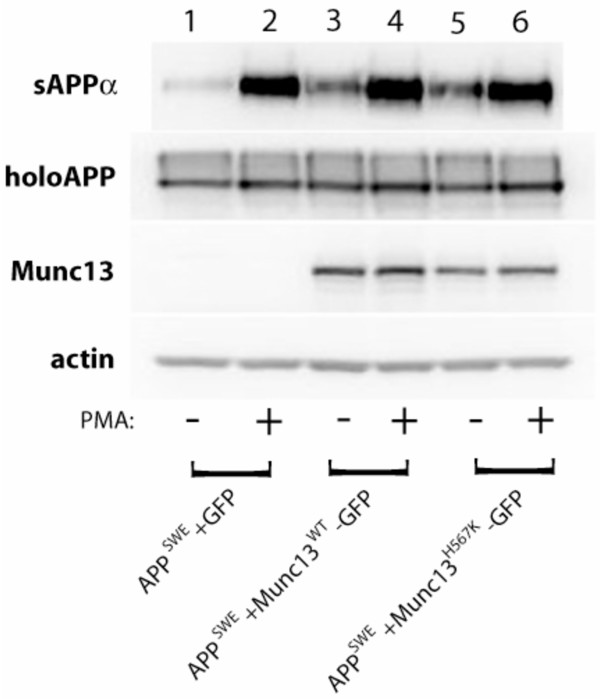
**Munc13-1 increases constitutive and phorbol-stimulated sAPPα secretion in a fashion that is independent of the integrity of its phorbol-sensing C1 domain**. Levels of soluble APPα (sAPPα) ectodomain were measured by Western blotting of cell supernatants with anti-APP antibody, 6E10, following the treatment of cells with DMSO (-) or 100 nM PMA (+) for 2 hours in a 37°C, 5% CO_2 _cell culture incubator. Levels of holoAPP were measured from cell lysates with anti-APP antibody 369. Levels of Munc13-1 wild type and Munc13-1 H567K mutant proteins were measured by anti-GFP antibody. Equal protein loading was verified by measuring the levels of actin protein in all cell lysates.

Introduction of either Munc13-1 wild type or Munc13-1 H567K mutant resulted in a significant, 3–5 fold increase in basal sAPPα release (Figure 1, lanes 1, 3, 5). Munc13-1 wild type and the Munc13-1 H567K mutant molecules were identical in their effects on basal (constitutive) sAPPα secretion (Figure 1, lanes 1, 3, 5). Since Munc13-1 is primarily a receptor for phorbol esters, which mimic the effects of endogenous diacylglycerol, we applied the phorbol ester PMA in the presence of either the wild type or mutant Munc13. Our results showed a typical increase in sAPPα release [29] in both Munc13-1 wild type and Munc13-1 H567K mutant transfected cells (Figure 1, compare lane 1 vs 2, lane 3 vs 4, and lane 5 vs 6; Table 1) indicating that phorbol ester interaction with histidine-567 of the C1 domain of Munc13-1 is not a key step in regulated shedding.

**Figure 2:**
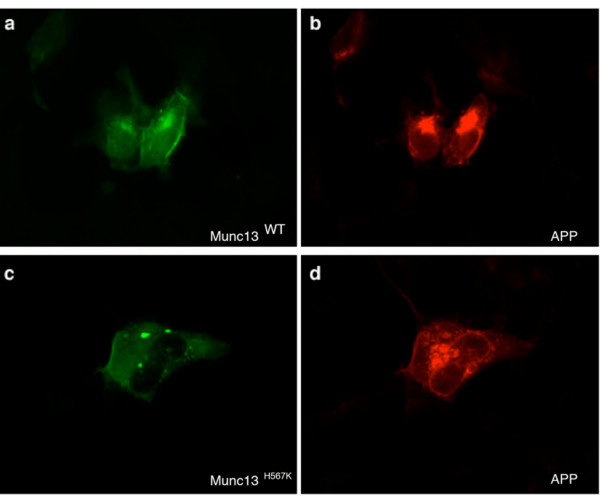
**Localization of APP following PMA treatment**. HEK293 cells were co-transfected with the following cDNAs: (a, b) APP^SWE^-pPrk5 and Munc13-1^WT^-pEGFP-N1 and (c, d) APP^SWE^-pPrk5 and Munc13-1^H567K^-pEGFP-N1. Treatment with 100 nM PMA was carried out for 2 hours. GFP immunofluorescence allowed visualization of (a) Munc13-1^WT ^and (c) Munc13-1^H567K ^mutant molecules (green). (b, d) APP was immunolabeled with rabbit polyclonal anti-APP-specific antibody 369 followed by rhodamine red conjugated secondary antibody (red).

The subcellular localization of APP was similar following phorbol ester treatment of cells transfected with either the wild type Munc13-1 or H567K Munc13-1 mutant (Figure 2, panels "b" and "d") whilst the characteristic difference in phorbol ester binding and intracellular localization between the wild type Munc13-1 and H567K Munc13-1 mutant was observed (Figure 2, panels "a" and "c"). As previously reported [11], in contrast to the wild type Munc13-1 (Figure 2, panel "a"), the H567K Munc13-1 mutant failed to translocate to the plasma membrane following phorbol ester application (Figure 2, panel "c").

Our evidence for the role of Munc13-1 in the constitutive sAPPα secretion in HEK293 cells is in agreement with recently published results by Roßner *et al*. that show increased basal sAPPα release from the brains of Munc13-1 wild-type mice compared with the brains from the Munc13-1 knock-out mice [13]. However, our results also demonstrate that the phorbol ester-sensing C1 domain of Munc13-1 is dispensable in the phorbol-stimulated shedding of APP from HEK293 cells. This is in contrast to the results reported by Roßner *et al*. who showed a marked reduction in the phorbol-regulated release of sAPPα in the presence of the phorbol-insensitve H567K Munc13-1 mutant in mouse neurons and in the human neuroblastoma cell line [13]. With regard to these contrasting results, we cannot exclude the possibility that our results disagree with those of Roßner *et al*. due to differences in experimental cell systems that were employed by each group to study the role of Munc13-1 in the APP metabolism (i.e., human neuroblastoma and wildtype APP in the case of Roßner vs HEK293 and Swedish APP in the current study). Of note, Munc13-1 apparently plays its usual role in constitutive sAPPα release (Figure 1, panels "a" and "e"), suggesting that Munc13-1-mediated vesicle priming is, at a first approximation, fully functional in HEK293 cells.

In reconciling our data, plus much published literature, together with the Munc13-1 data of Roßner, our best formulation is that there is redundancy at the level of effector molecules linking PKC/diacylglycerol signaling to APP shedding. In other words, in some circumstances, the effector seems highly likely to be phospho-state-sensitive since inhibition of protein phosphatases 1 and 2A with okadaic acid leads to the activated shedding phenotype. Okadaic acid would not be expected to interact with the C1 domain of Munc13-1, strengthening the case for the existence of a phospho-state-sensitive effector as opposed to a phorbol-sensitive nonphosphorylatable protein such as Munc 13-1. Though we were unable to confirm Roßner's data, because of the caveats mentioned above, we cannot definitively exclude the possibility that phorbols can activate APP shedding not only through a conventional PKC-dependent phospho-state-sensitive mechanism but also via a second, parallel, unconventional, PKC-independent, phospho-state-independent pathway involving Munc13-1.

Albeit nonparsimonious, this formulation does account for all the existing literature in this area. The existence of redundant mechanisms by which to transduce a wide variety of signals into altered rates of APP shedding suggests that the activated shedding phenomenon may be important for normal cellular or organ homeostasis. The best example of a known function for activated shedding involves leukocyte rolling in which selectins are used as anchors for cell motility at the leading edge of the advancing cell, but, once the cell has translocated, and the leading edge becomes the trailing edge, ectodomains must be released (shed) in order to permit forward movement to proceed. This comparison (among others) has been used to suggest that the normal function of APP may be in cell adhesion or cell-cell contact.

### Munc18

As discussed above, Munc18-1/Munc18-a/nSec/rbSec1 (Murine homologue of *S. cerevisiae *Sec1p and of *C. elegans unc18*) protein has been demonstrated to control multiple trafficking steps in the secretory pathway. These include 1) vesicle docking; i.e., attachment or tethering of vesicles to the plasma membrane (that occurs prior to the priming), 2) priming or "maturation" of docked vesicles ready for fusion, and 3) fusion of vesicles with the plasma membrane. Munc18 is a SNARE (**s**oluble ***N***-ethylmaleimide-sensitive fusion protein [NSF]-**a**ttachment protein [SNAP] **re**ceptors) complex accessory protein that participates in the regulation of neurosecretion by interacting with a SNARE protein syntaxin-1A [14,15]. Munc18 is phosphorylated by PKC on serines 306 and 313 in neuronal and neuroendocrine or chromaffin cells and the phosphorylation state of Munc18 regulates the spontaneous release of vesicular contents [16-18].

Munc18 would be predicted to interact with APP via adaptor proteins of the X11 family, two of which (X11α and X11β) contain Munc18 interacting domains (MIDs; [30]). These adaptors have been demonstrated to modulate trafficking and, consequently, processing of APP, resulting in an increased generation of sAPPα and decreased generation of Aβ [22,31,32]. Because APP interacts with Munc18 via X11, we analyzed the potential impact of wildtype and PKC-site phospho-mimetic mutant forms of Munc18 on APP processing in the absence or presence of X11. Serines at positions 306 or 313, or both, were changed to glutamic acid to generate the phospho-mimetic forms of Munc18.

**Figure 3:**
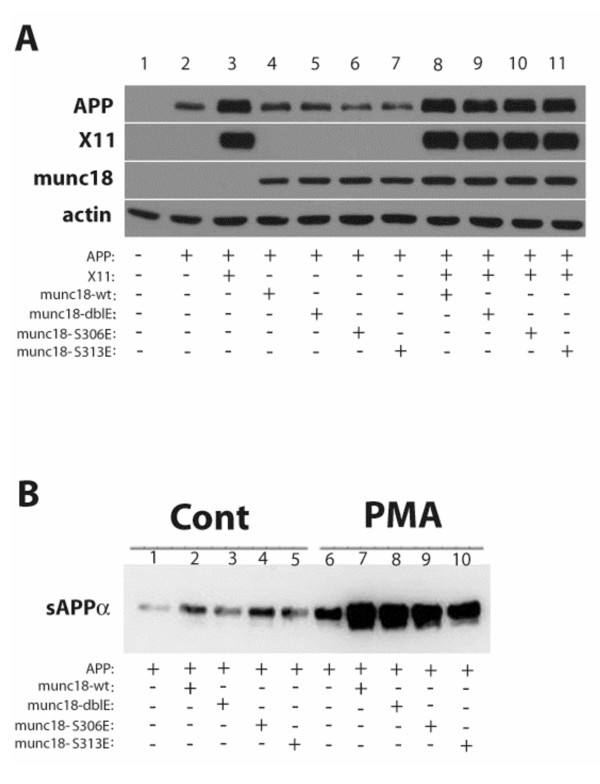
**APP metabolism and sAPPα release are not modulated by the phospho-state of Munc18 at serine 306, serine 313, or by dual phosphorylation at both residues**. (A) HoloAPP levels (top panel) were determined in the absence (lane 2) or presence of wildtype or phospho-site mutant forms of Munc18 (lanes 4–7) and in the presence of X11 alone (lane 3) or the combination of X11 plus each form of Munc18 (lanes 8–11). (B) Basal and PMA-stimulated sAPPα release were determined in the absence (lanes 1 and 6) or presence of wildtype (lanes 2 and 7) or phospho-site mutant forms of Munc18 (lanes 3–5 and 8–10).

**Figure 4:**
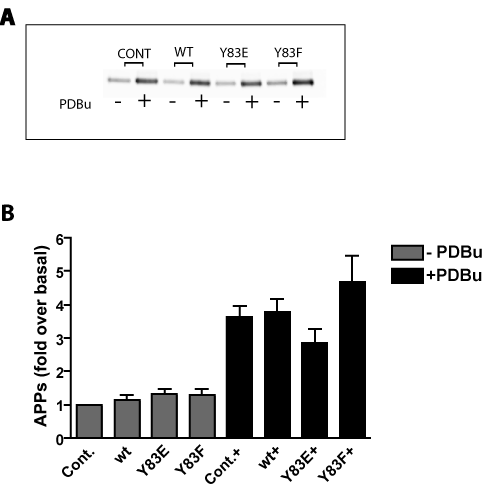
**Phorbol-stimulated sAPPα secretion is not sensitive to the phospho-state of NSF at tyrosine 83**. HEK293 cells stably expressing APP695 (HEK293-695) were transiently transfected with either wild type NSF (WT), the tyrosine 83 phospho state mimetic NSF mutant (Y83E), or the the tyrosine 83 dephospho state mimetic NSF mutant (Y83F). Empty vector was used as control (Cont). Cells were treated with PDBu for one hour after which media were collected and immunoprecipitated with antibody 6E10. Western blotting for sAPPα was performed using 6E10. (A) Representative Western blot for sAPPα in the absence (-) or presence (+) of PDBu. (B) Quantification of 3 such experiments.

Figure 3A shows immunoblots of the lysates of cells expressing various combinations of the molecules of interest. The presence of X11 appeared to stabilize the APP holoprotein, as reported [32]. No Munc18 Ser^306^- or Ser^313^-phospho-state-specific modulation of APP holoprotein levels was evident in the absence (lanes 4–7) or presence (lanes 8–11) of X11. Figure 4B shows that all forms of Munc18 enhanced sAPPα release, but sensitivity of sAPPα secretion to stimulation by PMA was observed regardless of the integrity or phospho-mimetic mutation of either or both Munc18 PKC phosphorylation sites. Further, since serine-to-glutamate (S-to-E) phospho-site mutants are designed to mimic constitutively phosphorylated amino acids, one might predicted that S-to-E phospho-site mutants would yield effects resembling those caused by phospho-forms of Munc18. In this case, one might predict that single S-to-E mutant forms of Munc18 or double S-to-E mutant Munc18 might enhance basal sAPPα release to an extent that would be greater than that of wildtype Munc18, but such an effect was not observed (Figure 4B). In some experiments, S-to-E double mutant Munc18 was associated with lower fold-effects in response to phorbol esters as compared with single mutant forms. This altered pattern appeared not to be attributable to phorbol sensitivity *per se*, but to a relative instability of the double mutant or enhanced sensitivity to proteolysis, as revealed by immunoblotting for Munc18 (data not shown).

### NSF

Recently, Huynh *et al*. identified a novel mechanism for regulation of vesicle fusion through the phosphorylation/dephosphorylation state of NSF at tyrosine 83 by the protein tyrosine phosphatase PTP-MEG2 [19]. The stoichiometry of NSF tyrosine phosphorylation in cells is very low, suggesting that this mechanism of vesicle fusion regulation is limited to a small subcellular secretory compartment. Huynh *et al*. proposed that PTP-MEG2 promotes secretory vesicle biogenesis or homeostasis by regulating the homotypic fusion of immature secretory vesicles, perhaps beginning with the first post-Golgi transport vesicles destined to become secretory vesicles [19]. PKC regulates α-secretase activity (and hence sAPPα levels) within the TGN [8]. It is possible that regulated sAPPα generation in the TGN may be due, at least in part, to regulation of fusion of APP-bearing vesicles with separate vesicles bearing α-secretase. To test this hypothesis we expressed wild type NSF (WT) or the tyrosine 83 substitution mutants Y83E and Y83F in HEK293 cells stably expressing APP695 (HEK293-695; [21]). Neither of the NSF constructs had any effect on either the basal or the PDBu-stimulated shedding of sAPPα (Figure 4) suggesting that the phospho-state of NSF does not control co-compartmentalization of APP transport vesicles and α-secretase transport vesicles or their fusion.

### Eve-1

Another candidate for phorbol ester receptors/PKC substrates are so called adaptor proteins that can modify α-secretase activity directly by binding to the ADAM proteases. A number of such adaptors have been identified using yeast two hybrid systems. These are Src homology 3 (SH3) domain-containing proteins that bind to proline-rich sequences in the cytoplasmic domains of ADAMs. Among these are endophilin I and SH3PX1, which bind ADAM9 and ADAM15 [33], p85α [34], Src [35,36], Grb [36] and Fish [37], which bind to ADAM12. More recently an additional adaptor, Eve-1, was identified using the cytoplasmic domain of ADAM12 as bait [20]. We were particularly interested in Eve-1 since it regulates the phorbol-stimulated shedding of pro heparin-binding EGF-like growth factor (HB-EGF), thus making it an excellent candidate as a PMES for APP. Furthermore, Eve-1 has been shown to interact with known α-secretases ADAMs 9, 10 and 17 in addition to ADAM12 [20].

**Figure 5:**
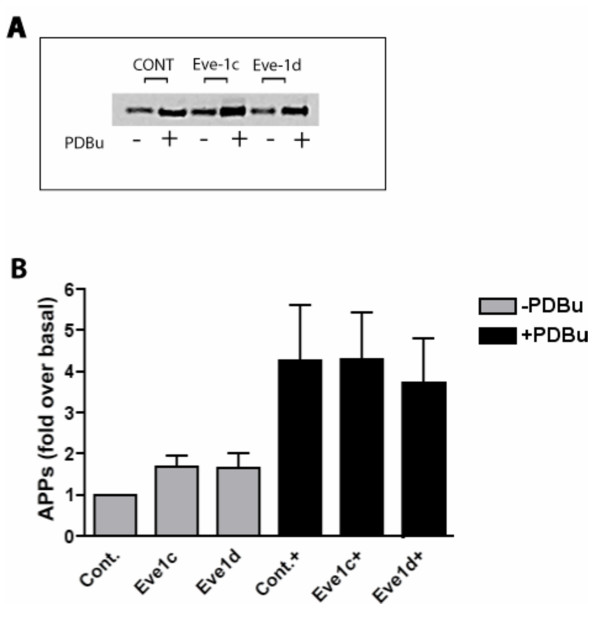
**Phorbol-stimulated sAPPα secretion is not modulated through Eve-1**. HEK293-695 were transiently transfected with either Eve1-b or Eve1-c. Empty vector was used as control (Cont). Cells were treated with PDBu and processed as above. (A) Representative Western blot for sAPPα in the absence (-) or presence (+) of PDBu. (B) Quantification of 3 such experiments.

We therefore tested the involvement of Eve-1 in phorbol-stimulated sAPPα generation by transiently expressing Eve-1-c and Eve-1-d (the two isoforms shown to bind ADAMs 9, 10 and 17) in HEK293-695 cells. We found no consistent effect of Eve-1c or Eve-1d on either basal or phorbol-stimulated sAPPα secretion (Figure 5), although, in some experiments, Eve-1c showed a nonsignificant trend toward potentiation of shedding. Further work on Eve-1 isoforms is ongoing. No ^32^PO_4 _incorporation into either Eve-1c or Eve-1d was detectable in response to phorbol ester treatment (not shown) despite the existence of consensus PKC phosphorylation sites. It is possible that separate adaptors exist that correspond to each protein that undergoes shedding (i.e., that Eve-1, the pro-EGF/ADAM adaptor, is not employed as an adaptor for APP/ADAM). To date, no adaptor has been identified that regulates α-secretase shedding of the APP ectodomain according to the phosphorylation state of the adaptor.

## Conclusion

The non-amyloidogenic processing of Alzheimer amyloid precursor protein is characterized by increased secretion of neurotrophic and neuroprotective soluble sAPPα species and by diminution of Aβ generation. The activation of the α-secretase pathway, that governs the non-amyloidogenic APP processing, represents one of the most important therapeutic targets for preventing and/or alleviating the Alzheimer's disease neuropathology. Indeed, a recent study by Postina *et al*. [38] has clearly demonstrated that overexpression of α-secretase ADAM10 leads to a reduction in amyloid burden in a mouse model of Alzheimer's disease [38]. In addition, the reduction in Aβ both *in vitro *and *in vivo *can be achieved through an activation of second messenger cascades, including protein phosphorylation. Activation of protein kinase C (with phorbol esters) [2,3,39], activation of protein kinase A (with forskolin) [40], inhibition of protein phosphatase 1 (with calyculin A) [41], and inhibition of protein phosphatases 1 and 2A (with okadaic acid) [4,41,42] are all intracellular signals that cause an increase in the release of non-amyloidogenic sAPPα and a concomitant decrease in Aβ when tested in continuous cell lines. In primary neuronal culture, the effect of okadaic acid on Aβ reduction is more substantial than the effect of phorbol esters [41], demonstrating that protein dephosphorylation pathways are especially important in regulating processing in neurons.

**Figure 6:**
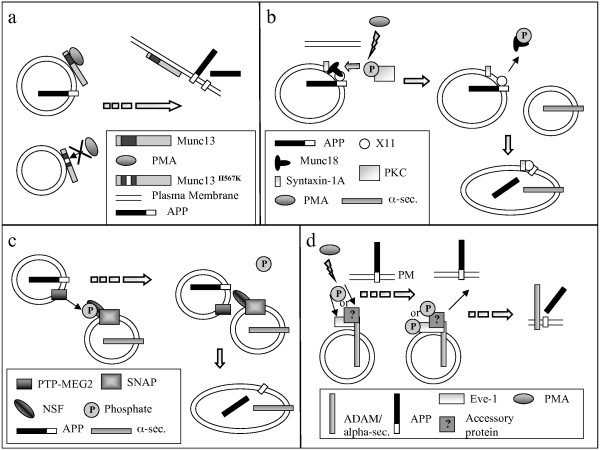
**Possible mechanisms for how the PMES candidates tested in this study might modulate shedding of the APP ectodomain**. a, This cartoon depicts how phorbol esters (PMA) might promote translocation of wildtype but not H567K Munc13-1 to the PM. b, This cartoon depicts how phosphorylation of Munc18 by PKC might facilitate fusion of APP transport vesicles with α-secretase transport vesicles, thereby facilitating shedding. c, This cartoon depicts how dephosphorylation of phospho-NSF might disinhibit NSF-mediated vesicle fusion; as in b, the notion is that APP and α-secretase might be traveling in separate vesicles prior to phosphorylation-state mediated facilitation of vesicle fusion. d, This cartoon depicts how the phosphorylation state of Eve-1 (or some theoretical accessory Eve-1 binding protein) might modulate vesicle interactions with the PM or they might modulate the physical docking between APP and α-secretase.

Because of the biological and potential clinical importance of regulated shedding, we embarked upon two strategies to elucidate its molecular basis. First, we identified the molecular machinery responsible for α-secretase cleavage in *Saccharomyces cerevisiae *[43]. In that case, endogenous α-secretase was identified as yapsin [44], a PI-linked intravesicular/cell surface protease rather than the integral metalloproteinases that catalyze the reaction in mammalian cells. Thus, this molecular diversity thwarted our strategy of discovering the basis for regulated shedding via a yeast genetics approach. In the current study, we took a "candidate molecule approach", examining four molecules that might plausibly underlie phorbol- or PKC-regulated APP ectodomain shedding. Potential mechanisms for how each of the four PMES candidates tested herein might act to regulate APP shedding are depicted in cartoon form in Figure 6; however, none of these four proteins appears to fulfill the essential criteria for a PMES.

The release of sAPPα and Aβ into the brain interstitium is dynamically regulated by neurotransmission and hormonal status [45-48] as a result of a series of phospho-state-sensitive reactions, including: (1) PKC-regulated intracellular α-cleavage at the TGN (8); (2) PKC-sensitive fission at the *trans*-Golgi network [10]; and (3) PKC-sensitive enzyme/substrate events intrinsic to the plasma membrane [49]. Further work will be required to identify the phospho-state-sensitive mediators of these reactions and to identify which reactions in the regulated ectodomain shedding process are most relevant to the pathogenesis, treatment, or prevention of the cerebral amyloidosis of Alzheimer's disease.

## Methods

### Materials

Phorbol 12-myristate 13-acetate (PMA), anti-actin antibody, streptavidin-linked horseradish peroxidase (HRP) antibody, IGEPAL (NP-40), Triton X-100 and other common chemicals were purchased from Sigma. APP-specific antibody 6E10 was purchased from Signet. Transfection reagent FuGENE, tablets containing a cocktail of protease inhibitors (Complete) and collagen were purchased from Roche Applied Science. Reagents for enhanced chemiluminescence assay (ECL) were purchased from Amersham Biosciences. Biotinylation reagent, Sulfo *N*-hydroxysulfosuccinimide ester (EZ-Link Sulfo-NHS-LC-Biotin) was purchased from Pierce. All cell media were obtained from Cellgro and all cell culture plastics was obtained from Nalgene, Nunc International. Rhodamine red- and Alexa Fluor 488-conjugated secondary antibodies were purchased from Invitrogen. Anti-Munc18 antibody was obtained from BD Transduction Laboratories. Mounting medium containing anti-fading agents was obtained from Biomeda. The pEGFP-N1 mammalian expression plasmid was purchased from Clontech. The pcDNA3 mammalian expression plasmid was purchased from Invitrogen.

### Cell lines

All experiments were performed in human embryonic kidney cells, HEK293. Untransfected cells were purchased from the American Type Culture Collection (ATCC). HEK293 cells over-expressing APP695 were described previously [21].

### Determination of soluble APP or sAPPα in cells expressing APP and Munc13-1

HEK293 cells were grown in 10 cm tissue culture dishes. Transfections were performed on 70–80% confluent cell monolayers with the following cDNAs: 1. 5 μg APP^SWE^-pPrk5 and 5 μg p-EGFP-N1; 2. 5 μg APP^SWE^-pPrk5 and 5 μg Munc13-1^WT^-pEGFP- N1; 3. 5 μg APP^SWE^-pPrk5 and 5 μg Munc13-1^H567K^-pEGFP-N1, by using FuGENE transfection reagent according to manufacturer's instructions. Munc13-1 mammalian expression plasmids were a gift from Dr Nils Brose, Max-Plank-Institute for Experimental Medicine, Goettingen, Germany. They contain either a full-length Munc13-1^WT ^(Munc13-1^WT^-pEGFP-N1) or a full-length Munc13-1 with a point-mutation, H567K, in a phorbol ester binding C1 domain (Munc13-1^H567K^-pEGFP-N1). Both the Munc13-1^WT ^and the Munc13-1^H567K ^mutant sequences are fused with a green fluorescent protein (GFP) at their C-termini, as described previously [11]. The pEGFP-N1 plasmid contains a sequence encoding only GFP and was used as a control plasmid in co-transfection experiments with the APP^SWE^-pPrk5 plasmid. Additionally, the pEGFP-N1 plasmid allowed the monitoring of transfection efficiencies between different experiments. 48 hours after the initiation of transfections, treatment of cells with phorbol 12-myristate 13-acetate (PMA) was carried out at a final concentration of PMA of 100 nM, in a serum-free medium, for 2 hours. Soluble APP or sAPPα was measured from collected cell culture medium by Western blotting with APP-specific antibody 6E10. Signal detection in all Western blotting experiments was carried out by using the enhanced chemiluminescence (ECL) assay. Visualization of signals and quantifications were performed by using LabWorks Imaging and Analysis Software (Ultra-Violet Products Bioimaging Systems).

### Cell surface content of APP

Following the collection of cell culture medium for sAPPα measurement, cell surface labeling of APP was carried out by using 0.5 mg/ml of a cell membrane-impermeable biotinylation reagent, Sulfo *N*-hydroxysulfosuccinimide ester (EZ-Link Sulfo-NHS-LC-Biotin). The biotinylation reagent was applied dissolved in ice-cold Dulbecco's Phosphate-Buffered Saline solution (DPBS) and incubated for 30 minutes on ice. The excess reagent was quenched with 50 mM Tris (pH 7.4). Cell lysates were prepared in phosphate-buffered saline (PBS)-containing 1% IGEPAL (NP-40) and a cocktail of protease inhibitors (Complete). Equal protein amounts were used for immunoprecipitation of APP with the APP-specific antibody 369. Western blotting and detection of cell-surface biotinylated APP were performed with streptavidin-linked HRP antibody.

### Determination of soluble APP or sAPPα in cells expressing APP and Munc18

HEK293 cells were grown in 6-well cell culture plates and transfected at 70–80% confluency with the following cDNAs, by using the FuGENE transfection reagent: 1. 1 μg APP^SWE^-pPrk5, 1 μg pcDNA3 and 1 μg pEGFP-N1; 2. 1 μg APP^SWE^-pPrk5, 1 μg Munc18wt-pcDNA3 and 1 μg pEGFP-N1; 3. 1 μg APP^SWE^-pPrk5, 1 μg Munc18dGlu-pcDNA3 and 1 μg pEGFP-N1; 4. 1 μg APP^SWE^-pPrk5, 1 μg Munc18S306E-pcDNA3 and 1 μg pEGFP-N1; 5. 1 μg APP^SWE^-pPrk5, 1 μg Munc18S313E-pcDNA3 and 1 μg pEGFP-N1. Munc18 plasmids have been described and used previously by Barclay *et al*. [16]. 100 nm PMA treatment of transfected cells was carried out approximately 48 hours following the start of transfections. Detection of soluble or sAPPα was performed from cell culture supernates by SDS-PAGE and Western blotting with anti-APP antibody 6E10.

### Measurement of holoAPP, Munc13 and Munc18 in cell lysates

Following the collection of cell culture medium for detection of sAPPα, cell lysates were prepared in lysis buffer containing 1% Triton X-100 and a cocktail of protease inhibitors (Complete) in phosphate-buffered saline (PBS). Levels of holoAPP were determined by using 8% Tris-glycine SDS-PAGE system and Western blotting with anti-APP antibody 369 [3]. Munc13-1 levels were detected by using anti-GFP antibody and Munc18 levels were detected with anti-Munc18 antibody. For verification of equal protein loading, Western blotting was also performed with rabbit polyclonal anti-actin antibody.

### Subcellular localization of APP, Munc13-1, and Munc18

HEK293 cells were transiently transfected, as described previously. 24 hours post transfections, cells were plated onto chamber slides coated with 2 mg/ml collagen. Following treatments with DMSO or 100 nM PMA, cells were fixed with 4% Paraformaldehyde in PBS for 10 minutes and then washed twice with PBS supplemented with Ca^2+ ^and Mg^2+ ^(PBS-CM) for 5 minutes per wash. For antibody uptake, cells were permeabilized with 0.5% Trition X-100 in PBS-CM at room temperature for 20 minutes. Non-specific binding of antibodies was blocked by incubating the cells with 10% bovine serum albumin (BSA) in PBS-CM for 30 minutes. For APP detection, APP-specific antibody 369 [3] was diluted at 1:300 in 5% BSA in PBS-CM and applied to cells at room temperature for 1 hour, followed by anti-rabbit rhodamine red-conjugated secondary antibody diluted at 1:400 in 5%BSA in PBS-CM also for 1 hour. Following three washes with PBS-CM and one wash with sterile deionized water, chamber walls were removed and cells were covered with a glass coverslip by using an aqueous mounting medium containing anti-fading agents. The immunofluorescence emitted from the GFP allowed visualization of Munc13-1^WT ^and Munc13-1^H567K^molecules. Munc18 was detected with anti-Munc18 antibody diluted at 1:300 in 5%BSA-PBS-CM at room temperature for 1 hour followed by anti-mouse Alexa Fluor 488-conjugated secondary antibody diluted at 1:400 in 5% BSA/PBS-CM for 1 hour. Specificity of protein localization for APP and Munc18 was confirmed by omitting primary antibodies from the labeling experiment. All fluorescently labeled molecules were detected with the Olympus BX51 fluorescent microscope.

### Determination of soluble APP or sAPPα in cells expressing APP and NSF or APP and Eve-1

HEK293 cells stably expressing APP695 (HEK293-695; [21]) were seeded onto 6-well plates at 1.5 × 10^5 ^cells per well. The following day, cells were transiently transfected with NSF and Eve-1 cDNAs using Fugene-6 (Roche) according to the manufacturer's protocol, and allowed to incubate for 48 hours. Empty vector was used as control. Cells were then treated either with 1 μM PDBu in DMSO or DMSO alone for 1 hour. Media were collected and immunoprecipitated with antibody 6E10. Precipitates were subjected to SDS-PAGE, blotted, and probed with 6E10. sAPPα levels were quantified by densitometry.

### Phosphorylation of Eve-1

Cells were cultured in a 35 mm dish and transfected with 2 μg of EVE-1 DNA for 48 hr. Prior to application of phorbol esters, cells were incubated in phosphate-free DMEM supplemented with 0.5 mCi/ml of ^32^P_i _for 2 hr. Cells were then treated with vehicle or with 1 mM PDBu for various intervals (0–45 min). At the end of treatment, cells were washed twice in PBS and lysed in RIPA buffer, containing 50 mM sodium fluoride, 200 mM sodium vanadate, and aprotonin. The lysate was spun for 15 minutes at 14,000 × g, and 180 ml of lysate were immunoprecipitated with 2.5 mg of anti-EVE antibody and Protein A. Precipitates were washed three times with PBS, and samples were boiled in sample buffer for 3 min, separated in a 7.5% polyacrylamide gel, and transferred to nitrocellulose and exposed to a phosphorimaging screen.

### Data analysis

Levels of sAPPα and surface APP, from seven independent experiments, were normalized against the levels of holoAPP (369 signal) and Munc13 levels (GFP signal), where appropriate, from the corresponding cell lysates. Grubbs' outlier tests were performed to assess and reject extreme values. The two group comparison of normalized values for sAPPα and surface APP, that represent comparison between HEK293 cells co-transfected with APP^SWE ^with a control plasmid and HEK293 cells co-transfected with either APP^SWE ^and Munc13-1^WT ^or APP^SWE ^and Munc13-1^H567K ^mutant, was performed by using a two-tailed *t *test. The same type of analysis was used to compare results obtained from HEK293 cells co-transfected with APP^SWE ^and Munc13-1^WT ^and from HEK293 cells co-transfected with APP^SWE ^and Munc13-1^H567K ^mutant. Differences were considered significant at a *p *value of < 0.01.

For experiments involving EVE-1 and NSF, levels of sAPPα secreted from HEK293-695 cells were quantified by densitometry. All sAPPα levels were normalized to basal secretion.

## Authors' contributions

AI, MC, SP, and LSB carried out all the experiments. JDB, TS, and SL participated in the design of the study and the writing of the manuscript. SH, TM, and RDB supplied reagents. SG conceived of the study and was the primary author of the manuscript. All authors read and approved the final manuscript.
